# Asymptomatic Shedding of Respiratory Virus among an Ambulatory Population across Seasons

**DOI:** 10.1128/mSphere.00249-18

**Published:** 2018-07-11

**Authors:** Ruthie Birger, Haruka Morita, Devon Comito, Ioan Filip, Marta Galanti, Benjamin Lane, Chanel Ligon, Daniel Rosenbloom, Atinuke Shittu, Minhaz Ud-Dean, Rob Desalle, Paul Planet, Jeffrey Shaman

**Affiliations:** a Department of Environmental Health Sciences, Mailman School of Public Health, Columbia University, New York, New York, USA; b Department of Biomedical Informatics, Columbia University, New York, New York, USA; c Sackler Institute of Comparative Genomics, American Museum of Natural History, New York, New York, USA; d Department of Pediatrics, Perelman School of Medicine, University of Pennsylvania, Philadelphia, Pennsylvania, USA; e Children’s Hospital of Philadelphia, Philadelphia, Pennsylvania, USA; University of Michigan—Ann Arbor

**Keywords:** asymptomatic infection, population health, respiratory viruses

## Abstract

Most observation of human respiratory virus carriage is derived from medical surveillance; however, the infections documented by this surveillance represent only a symptomatic fraction of the total infected population. As the role of asymptomatic infection in respiratory virus transmission is still largely unknown and rates of asymptomatic shedding are not well constrained, it is important to obtain more-precise estimates through alternative sampling methods. We actively recruited participants from among visitors to a New York City tourist attraction. Nasopharyngeal swabs, demographics, and survey information on symptoms, medical history, and recent travel were obtained from 2,685 adults over two seasonal arms. We used multiplex PCR to test swab specimens for a selection of common respiratory viruses. A total of 6.2% of samples (168 individuals) tested positive for at least one virus, with 5.6% testing positive in the summer arm and 7.0% testing positive in the winter arm. Of these, 85 (50.6%) were positive for human rhinovirus (HRV), 65 (38.7%) for coronavirus (CoV), and 18 (10.2%) for other viruses (including adenovirus, human metapneumovirus, influenza virus, and parainfluenza virus). Depending on the definition of symptomatic infection, 65% to 97% of infections were classified as asymptomatic. The best-fit model for prediction of positivity across all viruses included a symptom severity score, Hispanic ethnicity data, and age category, though there were slight differences across the seasonal arms. Though having symptoms is predictive of virus positivity, there are high levels of asymptomatic respiratory virus shedding among the members of an ambulatory population in New York City.

**IMPORTANCE** Respiratory viruses are common in human populations, causing significant levels of morbidity. Understanding the distribution of these viruses is critical for designing control methods. However, most data available are from medical records and thus predominantly represent symptomatic infections. Estimates for asymptomatic prevalence are sparse and span a broad range. In this study, we aimed to measure more precisely the proportion of infections that are asymptomatic in a general, ambulatory adult population. We recruited participants from a New York City tourist attraction and administered nasal swabs, testing them for adenovirus, coronavirus, human metapneumovirus, rhinovirus, influenza virus, respiratory syncytial virus, and parainfluenza virus. At recruitment, participants completed surveys on demographics and symptomology. Analysis of these data indicated that over 6% of participants tested positive for shedding of respiratory virus. While participants who tested positive were more likely to report symptoms than those who did not, over half of participants who tested positive were asymptomatic.

## Abstract (precis)

Respiratory viruses are common in human populations, causing significant levels of morbidity. Understanding the distribution of these viruses is critical for designing control methods. However, most data available are from medical records and thus predominantly represent symptomatic infections. Estimates for asymptomatic prevalence are sparse and span a broad range. In this study, we aimed to measure more precisely the proportion of infections that are asymptomatic in a general, ambulatory adult population. We recruited participants from a New York City tourist attraction and administered nasal swabs, testing them for adenovirus, coronavirus, human metapneumovirus, rhinovirus, influenza virus, respiratory syncytial virus, and parainfluenza virus. At recruitment, participants completed surveys on demographics and symptomology. Analysis of these data indicated that over 6% of participants tested positive for shedding of respiratory virus. While participants who tested positive were more likely to report symptoms than those who did not, over half of participants who tested positive were asymptomatic.

## INTRODUCTION

Respiratory virus infections are among the most common diseases in humans and cause significant morbidity and mortality across seasons and populations (1). While respiratory virus infections can often be mild, these infections are typically undocumented, as individuals may not seek medical care because either the infection is asymptomatic or it elicits symptoms not severe enough to warrant seeking care. As a consequence, standard medical surveillance provides an incomplete picture of the epidemiology of respiratory infections. In this study, which was an extension of a previously published study focusing on infection in this population during only the summer months (2), we focused on ascertaining the rates of asymptomatic infection for the following suite of respiratory viruses: adenovirus, coronavirus (CoV), human metapneumovirus (hMPV), human rhinovirus (HRV), influenza virus, respiratory syncytial virus (RSV), and parainfluenza virus (PIV). The population in this study was not only largely asymptomatic but also specifically ambulatory; we recruited participants from among visitors to a New York City tourist attraction. We therefore might have missed sampling severely symptomatic patients who were not well enough to visit the attraction, but we nonetheless can estimate the levels of prevalence in a setting where transmission is likely.

Some previous studies have measured or inferred asymptomatic respiratory virus shedding prevalence levels; however, such estimates range widely across demographics, pathogens, and sampling approaches. A study carried out in Nashville, TN, and Salt Lake City, UT, recruited 238 completely asymptomatic adults (no symptoms within 14 days of recruitment), who were controls for patients with pneumonia, from among visitors to outpatient primary care clinics for routine health checks. Of these individuals, only 5 (2%) tested positive for human rhinovirus (HRV), coronavirus (CoV), or human metapneumovirus (hMPV) (3). A different study, also based in Salt Lake City, UT, which prospectively recorded infection and symptoms over time within families recruited from the University of Utah campus community, found that 47% of respiratory infections among adults were asymptomatic. Influenza A virus, hMPV, CoV HKU1, and CoV OC43 were more frequently associated with symptoms (4). Among elderly adults enrolled in prospective surveillance in Rochester, NY, two studies have shown that very high proportions (80% to 90%) were symptomatic when infected with respiratory syncytial virus (RSV), influenza virus, and hMPV (5, 6).

Asymptomatic infection rates may vary as a function of host age and virus. In one study, 52% of asymptomatic children recruited from an Alaskan community tested positive for shedding respiratory virus in a one-time sampling scheme, with the majority having HRV and adenovirus (7). In another prospective surveillance investigation comparing positivity levels of symptomatic and asymptomatic infants in Perth, Australia, respiratory viruses were detected in 70% of samples from symptomatic infants and in 24.6% of samples from asymptomatic infants (8). In general, among children, studies have found that 24.6% to 64% of human rhinovirus (HRV) infections are asymptomatic (7–10). A number of studies have also documented the fraction of asymptomatic children who are shedding virus rather than the fraction of infected children who are asymptomatic. Calvo et al. documented an HRV shedding rate of 12.3% among healthy children (9). Van Benten et al. found that 20% of asymptomatic infants ≤2 years old tested positive for HRV (11). Similarly, Nokso-Koivisto et al. found that 20% of children without any past or recent respiratory infection symptoms tested positive for HRV or coronavirus (CoV) (12). Other studies examining multiple respiratory agents documented respiratory virus infection in 40% and 42% of children without symptoms (13, 14). The definition of symptomatic infection is not standardized, but most of these studies defined asymptomatic patients as those free from symptoms at the time of study participation or within a window around the time of enrollment.

This wide variation in symptomatic versus asymptomatic presentations underscores the need for further studies to understand the distribution of symptoms across infection statuses. Here, we document the shedding prevalence of a number of common respiratory viruses in an ambulatory adult population, as well as the distributions of symptomatology and symptom severity. Sampling was conducted in two seasonal arms: one carried out in the late spring/early summer (summer arm), which has been previously reported (2), and one carried out in the late winter/early spring (winter arm). In the summer arm, we found that over 5% of individuals tested positive for respiratory virus, with the majority testing positive for HRV, but with over half also reporting being asymptomatic. We hypothesized that these numbers might vary by season, as many respiratory infections, as well as ambulatory population contact dynamics, display seasonal patterns. (Since we published our previous study, GenMark Diagnostics, the manufacturer of the respiratory viral panel [RVP] assay used to analyze these samples, determined that a higher cutoff value for positivity was warranted for some of the assayed viruses. This paper thus rereports the summer study with a cutoff value of 25 nA/mm^2^ rather than 3 nA/mm^2^, so the numbers differ from those reported in Shaman et al. [2].)

## RESULTS

### Demographics.

We received consent from, surveyed, and swabbed a total of 2,685 individuals 18 years of age or older from among visitors passing through a room in the tourist attraction. Some directly approached the study recruiters, while others were present while a recruiter was explaining the study and elected to join afterward. A total of 1,477 participants were enrolled between 29 April and 31 July 2016 (summer) and a total of 1,208 between 28 January and 30 April 2017 (winter) (Table 1). Among all participants, 57.7% were reported as female, 41.7% as male, and 0.6% as transgender, gender nonconforming, or gender not known. Among the participants, 69.5% identified as white, 3.5% as Black/African American, 13.2% as Asian, 1.5% as American Indian/Alaskan Native, 0.6% as Native Hawaiian/Pacific Islander, 6.8% as other or of two or more races, and 4.9% gave no response. 21.7% identified as Hispanic, while 77.8% did not. A total of 39.8% of participants were in the 18-to-29-year-old age range, 20.9% were 30 to 39 years of age, 22.0% were 40 to 49 years of age, 12% were 50 to 64 years of age, and 4.2% were 65 or older. A total of 42.6% reported having had a flu shot, while 52.0% had not had one and 5.4% did not know whether they had had one. In response to the statement “I get sick more easily or more often than most people I know,” 3.4% reported that they strongly agreed, 11.3% somewhat agreed, 13.5% neither agreed nor disagreed, 23.0% somewhat disagreed, and 48.8% strongly disagreed. The breakdowns across all of the categorical variables described above were similar between the summer and winter arms of the study (see Table 1).

**TABLE 1:** Demographic characteristics of participants (total population and populations categorized by study arm)

Characteristic	Summer		Winter		All	
	*n*	%	*n*	%	*n*	%
All participants	1,477		1,208		2,685	
						
Gender						
Female	847	(57.4)	702	(58.1)	1,549	(57.7)
Male	617	(41.8)	502	(41.6)	1,119	(41.7)
Transgender	5	(0.3)	1	(0.1)	6	(0.2)
Gender nonconforming	6	(0.4)	2	(0.2)	8	(0.3)
Do not know	2	(0.1)	1	(0.1)	3	(0.1)
Race						
White	1,000	(67.7)	865	(71.6)	1,865	(69.5)
Black/African American	53	(3.6)	40	(3.3)	93	(3.5)
Asian	219	(14.8)	136	(11.3)	355	(13.2)
American Indian/Alaska Native	22	(1.5)	19	(1.6)	41	(1.5)
Native Hawaiian/Pacific Islander	5	(0.3)	11	(0.9)	16	(0.6)
Other or two or more races	87	(5.8)	95	(7.9)	182	(6.8)
Hispanic						
No	1,140	(77.2)	948	(78.5)	2,088	(77.8)
Yes	329	(22.3)	255	(21.1)	584	(21.7)
Age (yrs)						
18–29	568	(38.5)	486	(40.2)	1,054	(39.8)
30–39	309	(20.9)	252	(20.9)	561	(20.9)
40–49	301	(20.4)	290	(24.0)	591	(22.0)
50–64	206	(13.9)	117	(9.6)	323	(12.0)
65 or older	67	(4.5)	47	(3.8)	112	(4.2)
Flu shot in 2015–2016 season						
No	791	(53.6)	604	(50.0)	1,395	(52.0)
Yes	611	(41.4)	522	(44.1)	1,144	(42.6)
Do not know	58	(3.9)	69	(5.8)	146	(5.4)
Response to “I get sick more easily or more often than most people I know”						
Strongly agree	42	(2.8)	47	(3.9)	89	(3.4)
Somewhat agree	160	(10.8)	142	(11.8)	302	(11.3)
Neither agree nor disagree	178	(12.1)	180	(15.0)	358	(13.5)
Somewhat agree	341	(23.1)	271	(22.7)	612	(23.0)
Strongly disagree	741	(50.2)	554	(46.4)	1,295	(48.8)

### Viral positivity results.

**FIG 1:**
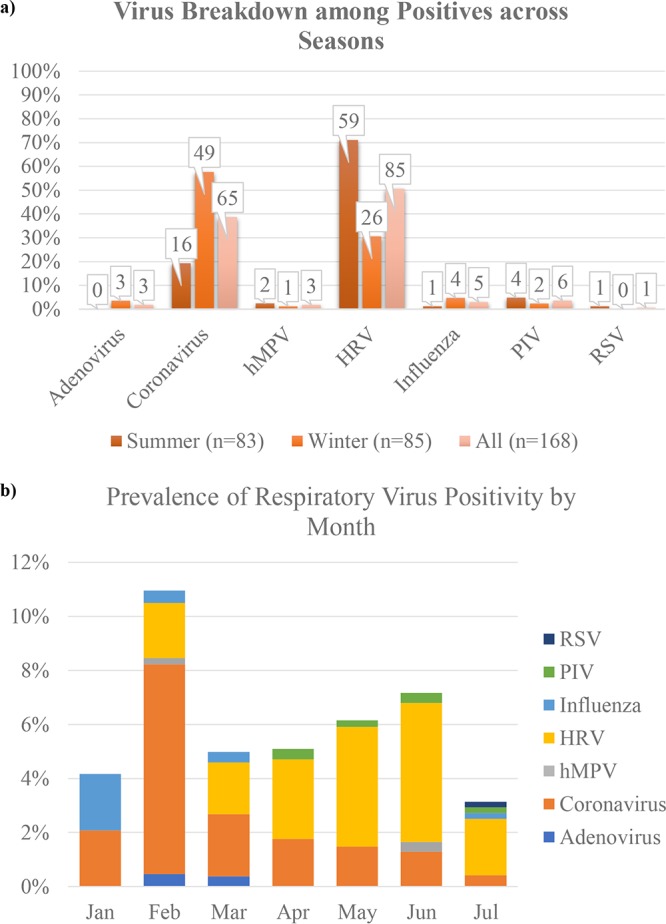
Virus breakdown among positives across seasons and by month. (a) Percentages of tests that were positive for each virus for the summer arm, the winter arm, and all participants. The numbers above each bar represent the absolute numbers of positive cases. (b) Prevalence of total positive tests by month by virus.

Among all 2,685 participants across both seasons, there were a total of 168 (6.2%) individuals who tested positive for respiratory virus. Of these, 85 (50.6%) were positive for HRV, 65 (38.7%) for CoV, and 18 (10.2%) for adenovirus, hMPV, RSV, influenza virus, or PIV. There were no coinfections. In the summer arm, only 5.6% of participants tested positive (2), whereas in the winter arm, 7.0% did. These positivity results did show differences across seasons in terms of virus distribution (Fisher’s exact test *P* = <0.001) though not in terms of overall positivity prevalence (*P* = 0.13). The majority of infections in the summer arm were HRV infections (71.0%), with 19.3% testing positive for CoV and the other 9.7% testing positive for influenza virus, hMPV, RSV, or PIV; however, the majority of infections in the winter arm were CoV infections (57.6%), with 30.6% testing positive for HRV and the remaining 11.8% testing positive for adenovirus, hMPV, influenza virus, or PIV (Fig. 1a). There was evidence of differing viral positivity rates across the months, with the highest proportion of positive tests (11.1%) occurring in February (*P* < 0.001). Figure 1b shows the percentage of positive tests per month broken down by virus.

### Analysis of positivity.

Testing positive for respiratory virus was positively associated with consumption of cold and flu medicine among all participants, and this association was consistent across the seasonal arms (Fisher’s exact test *P* = <0.001). The proportion of participants testing positive who were symptomatic ranged from 3.6% to 39.4% overall, depending on symptomatic definition. There were slight differences between the seasonal arms, with 2.5% to 48.1% of positives being symptomatic during the summer arm and 4.8% to 30.1% being symptomatic for the winter arm, again depending on definition. There was some evidence of differences in the proportions of infections that were symptomatic between seasons by symptomatic definitions 1 and 2 (chi-square *P* = 0.05 and 0.03, respectively) (see Materials and Methods for an explanation of the scoring method used for quantification of symptomatic definitions), but there was no evidence of differences for any other definition. Rates of being symptomatic differed significantly among virus-positive and virus-negative participants, with positive-testing participants more likely to meet symptomatic criteria, though there were up to 17.6% of individuals who tested negative who were nonetheless symptomatic (depending on definition) (Table 2). This association was consistent across the seasonal arms. No other univariate associations between testing positive for respiratory virus and other collected characteristics (e.g., race, gender, age, etc.) were found.

**TABLE 2:** Analysis of differences in proportion symptomatic by viral positivity (total population and populations categorized by study arm)

Definition	% positive		% negative		*X*^*2*^ value	*P* value	Fisher’s exact test value	Odds ratio	Confidence interval
	Symptomatic	Asymptomatic	Symptomatic	Asymptomatic					
Summer									
1	45.7	54.3	13.2	86.8	62.96	<0.01		5.52	3.4–8.8
2	48.1	51.9	17.1	82.9	47.89	<0.01		4.48	2.8–7.1
3	23.5	76.5	5.8	94.2	37.66	<0.01		5.00	2.8–8.7
4	2.5	97.5	0.4	99.6	5.62	0.02	0.07	5.64	0.5–32.2
5	8.6	91.4	1.6	98.4	18.86	<0.01	<0.01	5.68	1.9–14.3
6	2.5	97.5	0.5	99.5	4.64	0.03	0.09	4.84	0.5–26
7	7.4	92.6	1.3	98.7	17.04	<0.01	<0.01	5.89	1.9–16.1
									
Winter									
1	29.8	70.2	14.6	85.4	13.45	<0.01		2.47	1.5–4
2	31.0	69.0	18.1	81.9	8.36	<0.01		2.03	1.2–3.3
3	14.3	85.7	6.6	93.4	6.84	<0.01		2.36	1.2–4.4
4	4.8	95.2	0.5	99.5	16.55	<0.01	0.01	9.07	1.8–39.1
5	8.3	91.7	2.0	98.0	13.08	<0.01	<0.01	4.44	0.7–11.2
6	4.8	95.2	0.6	99.4	14.41	<0.01	0.01	7.77	1.6–31.3
7	4.8	95.2	1.6	98.4	4.17	0.04	0.06	3.00	0.7–9.4
									
All									
1	37.6	62.4	13.9	86.1	66.88	<0.01		3.74	2.7–5.2
2	39.4	60.6	17.6	82.4	47.87	<0.01		3.05	2.2–4.2
3	18.8	81.2	6.2	93.8	37.93	<0.01		3.53	2.3–5.3
4	3.6	96.4	0.5	99.5	22.34	<0.01	<0.01	7.64	2.3–22.3
5	8.5	91.5	1.8	98.2	31.81	<0.01	<0.01	5.06	1.9–9.7
6	3.6	96.4	0.6	99.4	19.09	<0.01	0.01	6.55	2–18.4
7	6.1	93.9	1.5	98.5	18.81	<0.01	<0.01	4.31	1.9–9.1

The best-fit logistic regression models according to minimum Akaike information criterion (AIC) values are shown in Table 3. For all participants combined across seasons, the best-fit model supported an association of an increased likelihood of testing positive for respiratory virus infection (all viruses) with a higher total symptom score but with no other variables. There was some difference between the arms, with the association for the summer arm holding for the symptom score and being Hispanic and that for the winter arm holding only for the symptom score. Performing the analysis on all participants (both arms) for HRV positivity, the best-fit model showed an association of positivity with symptom score, being Hispanic, and age category. For the summer arm and winter arm considered individually, the association held only for symptom score and being Hispanic. For CoV positivity, the best-fit model indicated an association between positivity and symptom score for both seasonal arms.

**TABLE 3:** Best-fit logistic regression models describing virus-positive status as a function of demographic variables and self-reported symptomology^a^

Characteristic	All virus		Rhinovirus		Coronavirus	
	Odds ratio (95% CI)	*P* value	Odds ratio (95% CI)	*P* value	Odds ratio (95% CI)	*P* value
Summer						
Symptom score	1.26 (1.19–1.34)	<0.001	1.25 (1.17–1.33)	<0.001	1.27 (1.13–1.42)	<0.001
Hispanic						
No	Ref	Ref	Ref	Ref		
Yes	1.67 (1.0–2.72)	0.04	1.72 (0.95–3.03)	0.06		
						
Winter						
Symptom score	1.14 (1.08–1.20)	<0.001	1.15 (1.05–1.25)	<0.001	1.1 (1.01–1.18)	0.02
Hispanic						
No			Ref	Ref		
Yes			1.94 (0.82–4.36)	0.11		
						
All						
Symptom score	1.19 (1.15–1.24)	<0.001	1.20 (1.14–1.27)	<0.001	1.15 (1.08–1.22)	0.001
Hispanic						
No			Ref	Ref		
Yes			1.65 (1.02–2.63)	0.04		
Age category						
18–29			Ref			
30–39			2.07 (1.2–3.54)	0.008		
40–49			1.1 (0.58–2.04)	0.75		
50–64			1.04 (0.41–2.3)	0.93		
65+			0.45 (0.02–2.15)	0.43		

a Separate models are presented for positivity for any virus, human rhinovirus (HRV) only, and coronavirus (CoV) only. CI, confidence interval; Ref, reference value.

### Analysis of quantitated signal intensity score.

Among the positive-testing participants, signal intensity (measured continuously in nanoamps per square millimeter) was positively associated with reporting allergies (*P* < 0.05). There were differences between the seasonal arms for this finding, however. For the winter arm, allergies were similarly associated with quantitated score of signal intensity for all positives, whereas there was an association of signal intensity with age but not with allergies for the summer arm (there was a negative association between signal intensity and the 40-to-49-year-old and 50-to-64-year-old age categories compared to the 18-to-29-year-old age category). Among HRV positive-testing participants (both arms combined), there was strong evidence of a negative association between signal intensity and the 50-to-64-year-old age category compared to the 18-to-29-year-old age category, weak evidence of a positive association between signal intensity and having allergies, and weak evidence of a positive association between signal intensity and being Hispanic. Again, there were seasonal differences, with the symptom score being negatively associated with signal intensity in the winter arm and with being Hispanic being positively associated in the summer arm. For those positive for CoV, signal intensity was weakly positively associated with having allergies (*P* < 0.1). This association held across seasons, with stronger association in the winter arm (*P* = 0.02). In the summer arm there were also associations with symptom score, race, and gender, with signal intensity being positively associated with symptom score, a negative association between signal intensity and all other races compared to whites, and a negative association with women compared to men.

### Symptom results.

**FIG 2:**
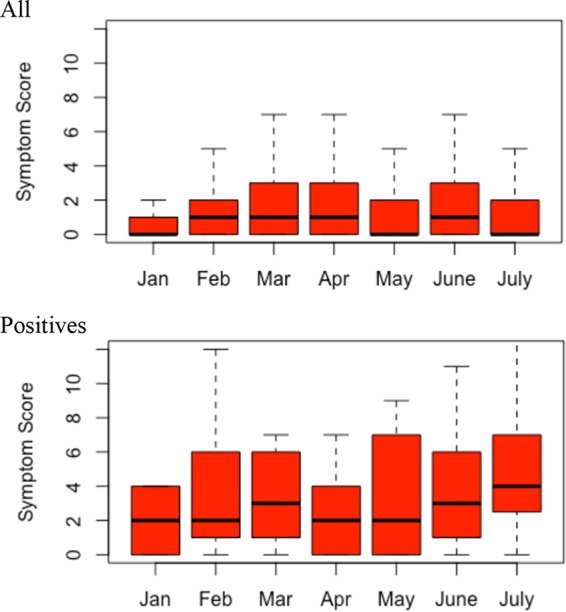
Symptom scores by month. This figure shows mean symptom scores by month among all participants (top) and among the participants testing positive (bottom). There were statistically significant differences in mean symptom scores by month among all participants (*P* < 0.001 [ANOVA]) but not among only those testing positive (*P* = 0.914 [ANOVA]), indicating that it is likely that higher respiratory virus prevalence during some months explains the variations in symptom scores over seasons. Note that the sampling in January did not start until near the end of the month, so the sample size was small and may not reflect the true mean symptom scores.

Among all participants, there was strong evidence of a higher reported total symptom score among women than men (analysis of variance [ANOVA] *P* < 0.001); participants aged 30 to 39 years, 40 to 49 years, and 50 to 64 years had lower total reported symptom scores than participants aged 18 to 29 years (ANOVA *P* < 0.001). These results were consistent and significant across the seasonal arms. Reporting consumption of cold and flu medicines was positively associated with higher total symptom scores across all participants and also only among positives (ANOVA *P* < 0.0001). This finding was also consistent across seasons. There was evidence of differences in symptom scores by month across all participants (ANOVA *P* < 0.001), but there were no differences seen in assessing only the participants testing positive (AOV, *P* = 0.725) (Fig. 2). There were, however, differences in mean individual symptom scores by virus as shown in Table 4.

**TABLE 4:** Mean symptom score for each symptom among individuals testing positive for each virus

Virus	Symptom score								
	Fever	Chills	Muscle pain	Cough	Chest pain	Sneeze	Sore throat	Watery eye	Runny nose
Adenovirus	1.0	1.0	1.0	1.0	0.7	1.0	1.0	0.7	1.0
Coronavirus	0.1	0.1	0.3	0.2	0.0	0.7	0.2	0.4	0.9
hMPV	0.7	0.7	0.7	1.7	0.7	1.0	0.7	0.7	1.0
HRV	0.1	0.1	0.4	0.6	0.0	0.8	0.5	0.4	0.9
Influenza virus	0.2	0.0	0.2	0.6	0.0	0.6	0.4	0.2	0.6
PIV	0.0	0.0	0.0	0.8	0.0	0.2	0.6	0.0	0.4
RSV	0.0	0.0	0.0	0.0	0.0	2.0	0.0	2.0	3.0

There was a positive association between reporting a greater tendency to get sick and total self-reported symptom scores among all participants (AOV, *P* < 0.0001); however, there was no significant association between viral positivity and reporting a greater tendency to get sick. These results were consistent across the seasonal arms.

Interestingly, the proportions of symptomatic individuals (by the various definitions) who tested negative were quite high (66.7% to 86.9%), and this finding held across the seasonal arms. Examining this result further, we found that for symptom definitions 1 and 2 (the definitions that encompassed all symptoms), there was a strong association among the participants who tested negative between being symptomatic and having allergies (*P* < 0.001). The association between having allergies and being symptomatic among participants who tested negative was less strong for definition 2 (*P* < 0.05) and nonexistent for definitions 3 to 7.

## DISCUSSION

In this two-part study, we found that 6.2% of adult visitors to a New York City tourist destination tested positive for the shedding of respiratory virus, with 5.6% testing positive in the late spring and summer months and 7.0% testing positive during the winter and early spring months. Regardless of symptom definition, over half of the participants who tested positive could be categorized asymptomatic, across seasons, whereas 81.9% to 99.6% of participants who tested negative were categorized asymptomatic. Prior estimates of symptomatic rates for persons positive for respiratory virus ranged from ~9% to 80% (9, 10, 15, 16). The results from this study determined on the basis of less-strict definitions 1 and 2 were consistent with those estimates. However, the results based on symptom definitions 3 to 7, which incorporated stricter definitions, indicated a lower percentage of symptomatic individuals than had been seen in some previous studies.

The winter results presented in this paper largely confirm the findings reported in the previously published summer study, with some slight differences in seasonal prevalence and breakdown of infection composition. For the summer arm, the winter arm, and the entire study, respectively, 25.9%, 32.8%, and 29.6% of individuals testing positive reported no symptoms at all (symptom score of zero). These numbers indicate that though there may be a spectrum of symptom severity, a substantial number of infected individuals are completely asymptomatic. Regardless, the participants in this study, even if symptomatic by the definitions used (see Materials and Methods), felt well enough to visit a tourist attraction. There were few individuals who reported severe symptom scores or fever, indicating that those with severe symptomatology might have stayed home and thus were not captured in this study.

Conversely, a substantial percentage of individuals who were symptomatic across the definitions did not test positive. Only some of the variation appears to have been due to allergies and only for symptom definitions 1 and 2. This result may indicate that symptoms persist after an infection has cleared, that symptoms may appear before an infection is detectable, or that symptoms may be due to infections not tested for in this study.

In multivariate analysis of viral positivity, we found an expected association between having worse symptoms and testing positive for respiratory virus. We also found some evidence of an association of Hispanic ethnicity with being positive, in particular, for HRV. This association has been found previously in other studies of respiratory virus (17, 18), especially in children. Some of the best-fit models also included the age category, although the only statistically significant result was that individuals aged 30 to 39 years were more likely to be positive than individuals aged 18 to 29 years. This association might be due to the fact that 30-to-39-year-old adults may be more likely to have or to be in contact with young children, who classically have a high prevalence of respiratory virus infection and frequently transmit infection to their parents (M. Galanti, R. Birger, S. M. M. Ud-Dean, I. Filip, H. Morita, D. Comito, S. Anthony, G. A. Freyer, S. Ibrahim, B. Lane, C. Ligon, P. Planet, R. Rabadan, A. Shittu, E. Tagne, J. Shaman, submitted for publication). However, this association could also have manifested from selection bias represented by the individuals who chose to participate in the study.

Manufacturer specifications, as well as data from previous studies, indicate that there is no association between eSensor signal intensity and the amount of virus present in a sample. However, in this study, we found evidence of an association between signal intensity and having allergies among all positives combined from both study arms and also among those in the winter arm alone (although not among those in the summer arm alone). There was, however, some association between signal intensity and age in the summer arm.

These findings build on our previously reported summer arm study and provide an estimate of baseline prevalence of respiratory virus shedding in an ambulatory population across seasons. The results indicate that nearly 1 in 17 adults is shedding respiratory virus across seasons and that nearly 1 in 14 do so during the peak winter month. There is evidence of a difference in virus species prevalence among seasons, with HRV being the most prevalent virus species during the summer arm (more than half of all reported summer infections were HRV infections) and coronavirus being dominant during the winter arm (more than half of all winter infections reported were coronavirus infections). The impact of shedding prevalence on contagiousness or transmission is not yet well defined, however.

There are several limitations to this study. While the sampling scheme was designed to capture individuals from a population different from those represented in studies of persons seeking medical attention, it would not capture individuals who stay home with severe symptoms. However, given that the site used in the study is frequented by tourists, who usually only have a limited amount of time in New York City, we hypothesize that many people might still visit the site despite having symptoms that would otherwise keep them home. Consequently, our sample may be less skewed in symptom distribution than a random sample from the street. Conversely, among tourist attraction visitors, those with symptoms might have been more inclined to participate in this study. Symptoms were self-reported, which can introduce error, and were reported only for the previous 48 h. For some viruses, RNA can be detected for several weeks following infection, so some seemingly asymptomatic individuals who tested positive may have been previously infected and symptomatic. Further, shedding prior to symptom development also occurs (19). These dynamics preclude calculation of the proportion of infections that are asymptomatic, but not estimation of asymptomatic prevalence in the ambulatory population, as the participants were asymptomatic at the time and were still shedding. Also, the study was run over two seasons, but there were no samples taken during the late summer or fall or early winter season, when prevalence levels may be different. For the diagnostic analysis, we performed a molecular test but did not try to isolate viruses or determine titers and therefore cannot confirm whether the virus collected in samples was viable. Lastly, this study excluded children, who have a high burden of respiratory virus infection.

The findings presented here indicate a significant level of respiratory virus shedding in an ambulatory adult population across seasons, as well as a substantial proportion of infections that are asymptomatic. These results could help improve estimates of virus incidence and inform disease transmission modeling and forecasting, which could then be used to support control efforts. Indeed, determination of respiratory virus prevalence in nonclinical settings can help with the design of control measures in the real-world settings where most transmission occurs. Future potential work in this area could include replicating this study in other highly frequented areas in the city (e.g., subway stations and parks) and enrolling children in order to obtain estimates across a broader age range.

## MATERIALS AND METHODS

As described in a report of a previous study (2) for which this study served as an extension, participants were recruited from a New York City tourist site frequented by both New York residents and tourists, thus providing a representative sample of visiting and local populations. Two rounds of participant recruitment were undertaken: the first from 29 April to 31 July 2016 (summer) (2) and the second from 28 January to 30 April 2017 (winter). All parts of the recruitment process (i.e., participant solicitation, consenting, surveying, and sampling) were performed during weekend days. Participants had to be over 18 to enroll and had to provide informed consent after reading a detailed description of the study (CUMC IRB AAAQ4358; AMNH IRB FWA00006768). Consented individuals were given a baseline survey, and one nasopharyngeal swab sample was collected from each nostril.

### Survey.

Participants were surveyed with respect to demographic attributes, including age, gender, and race, as well for as other information, including reports of recent travel, preexisting medical conditions, allergies, self-reported propensity to get sick, and flu shot status. Data were also collected, per the common cold questionnaire (20), on whether participants were experiencing symptoms (over the previous 48-h period) commonly related to respiratory infection: fever, chills, muscle pain, watery eye, runny nose, sneezing, sore throat, cough, and chest pain. The severity level for each symptom was recorded on a Likert scale (none, mild, moderate, and severe; scored at 0, 1, 2, and 3, respectively), and a total symptom score was calculated by adding together the values for all 9 symptoms.

### Specimen collection and analysis.

Nasopharyngeal swabs from both the left and right nasal cavities were collected from each participant using a Minitip flocked swab (Copan Diagnostics, Murrieta, CA). The samples were jointly stored in 2 ml DNA/RNA Shield (Zymo Research, Irvine, CA) for up to 30 days at 4 to 25°C and were subsequently divided into 2 aliquots and stored at −80°C until assay. Nucleic acids were extracted from 200-µl samples, with the addition of 10 µl of MS2 bacteriophage as an internal control, using an easyMAG NucliSENS system (BioMérieux, Durham, NC). Samples were tested for infection using a multiplex PCR assay known as an eSensor XT-8 respiratory viral panel (RVP; GenMark Diagnostics, Carlsbad, CA) according to the manufacturer’s instructions.

Positive samples were identified using a threshold of 25 nA/mm^2^. The viruses detected by the XT-8 RVP include influenza A (any subtype), A/H1N1, A/H3N2, and AH1N1pdm2009 virus and influenza B virus; RSV A and B; PIV 1, 2, 3, and 4; hMPV; HRV; adenovirus B/E and C; and CoV 229E, NL63, OC43, and HKU1.

### Definitions of asymptomatic infections.

There is no standard definition of symptomatic infection, so several definitions were used so that the sensitivity of our findings could be tested. All definitions were based on self-reporting of symptom type and severity. Definition 1 consisted of reporting at least two symptoms, with at least one being moderate or severe (21). Definition 2 required only one moderate to severe symptom. Definitions 3 to 7 were based only on fever, cough, and sore throat symptom reporting, as used to define influenza-like illnesses (22). Definition 3 required at least one of those three symptoms to be moderate to severe. Definition 4 required moderate to severe fever and moderate to severe cough or sore throat. Definition 5 required mild to severe fever and mild to severe cough or sore throat. Definition 6 required moderate to severe fever and mild to severe cough or sore throat. Lastly, definition 7 required mild to severe fever and moderate to severe cough or sore throat.

### Statistical analysis.

We conducted a range of statistical tests to identify associations between various demographic factors and both symptom scores and viral positivity. For categorical variables, such as gender and age group, we used ANOVA and Tukey tests to determine whether there were statistical differences in the average symptom scores between categories of the variable. We used chi-square and Fisher’s exact tests to compare differences across each categorical variable with respect to positivity. We performed univariate and multivariate regression to test associations between each set of variables (race, gender, age group, allergies, travel, residence, and Hispanic self-identification) and quantitated positivity scores (measured in nanoangstroms per square millimeter) for all viruses and for HRV and CoV separately. Logistic regression was used to conduct a similar analysis on associations between the demographic variables and positivity as a binary variable (positive versus negative) for all viruses and for HRV and CoV separately. The Akaike Information Criterion was used to identify the best-fitting models.
