# Correction for Birger et al., “Asymptomatic Shedding of Respiratory Virus among an Ambulatory Population across Seasons”

**DOI:** 10.1128/mSphere.00667-18

**Published:** 2018-12-12

**Authors:** Ruthie Birger, Haruka Morita, Devon Comito, Ioan Filip, Marta Galanti, Benjamin Lane, Chanel Ligon, Daniel Rosenbloom, Atinuke Shittu, Minhaz Ud-Dean, Rob Desalle, Paul Planet, Jeffrey Shaman

**Affiliations:** a Department of Environmental Health Sciences, Mailman School of Public Health, Columbia University, New York, New York, USA; b Department of Biomedical Informatics, Columbia University, New York, New York, USA; c Sackler Institute of Comparative Genomics, American Museum of Natural History, New York, New York, USA; d Department of Pediatrics, Perelman School of Medicine, University of Pennsylvania, Philadelphia, Pennsylvania, USA; e Children’s Hospital of Philadelphia, Philadelphia, Pennsylvania, USA; f Earth Institute, Columbia University, New York, New York, USA

## AUTHOR CORRECTION

Volume 3, no. 4, e00249-18, 2018. https://doi.org/10.1128/mSphere.00249-18. Page 1: Ruthie Birger should have two affiliations, as shown in this author correction.

